# Is It English?

**Published:** 1891-12-26

**Authors:** 


### The Hospital

A WEEKLY JOURNAL OF

**Science, Medicine, Nursing, and Philanthropy.**

*For Hospitals, Asylums, Infirmaries, Dispensaries, Medical Practitioners, Students, Nurses, and the Charitable Public.*

VOL. XI.—No. 274.] [DEC. 26, 1891.

### IS IT ENGLISH?

THE spirit, the mental essence of the typical Englishman is peculiar. It differs much from that, for example, of the Chinaman, and, indeed, from that of the typical man of every other race. It is on the whole a noble spirit, capable of doing things that are both worthy and effective. There is, perhaps, not a nobler spirit in any race of men. Such a spirit, such a mental essence is well worth preserving. The Press, both the book and the newspaper Press, inevitably reveals the spirit of the writers of the day; and as inevitably acts upon the minds of those who read. Any man who really understands what is going on about him, and who desires to preserve and to make nobler the typical English spirit, cannot help regarding with constant anxiety the tone and character of the Press.

On Friday, December 18, the *Pall Mall Gazette*, in a leading article devoted to the consideration of General Booth's social work during 1891, sought to exalt the merit and value of that work by speaking evil of the work of hospitals. The *Pall Mall* stated that "If this is the year of 'Darkest England,' it is also the year in which 'More Hospital Scandals' has become a familiar heading in the press, and which has shown us a blind and fatal obscurantism on the part of hospital officials."

Now we will not pay the *Pall Mall* writer the sorry compliment of supposing either that he does not know what he means, or that he does not mean what he says. Being an intelligent man and a practised journalist, he must be aware of the full significance of his words; and being a just man and a kindly, it is not to be believed that he would make damning charge against the hospitals at Christmas time and so deprive hundreds of workmen of the possibility of securing skilled medical attendance and good nursing in dangerous illness during the coming year, without considering what he was doing. But if it be the fact that during the year the phrase, "More Hospital Scandals" has become a familiar heading in the Press, and if it be true that the year 1891 "has shown us a blind and fatal obscurantism on the part of the hospital officials," then the *Pall Mall*, in the interests of the sick and suffering poor is bound, as the least thing it can do, to make good its charges by a detailed statement of particulars.

This is a question which seriously affects half a million poor hospital patients. But before dealing with the subject as it affects hospital patients is it not a right and proper thing to ask that justice shall be done even to hospital officials? A right-minded Englishman will not blacken the character of another Englishman, or of a man of any sort, without a full knowledge of the facts on which the blackening depends. And if it be not either right or reasonable to blacken the character of one man without sufficient cause, why is it right and reasonable to blacken the character of ten men, or of a hundred, or of a thousand?

There are persons who are responsible and persons who are irresponsible. A Billingsgate fish-wife may say things in a rage, which contain a grain of truth with a shipload of exaggeration. Nobody ever takes any notice of her because she is a Billingsgate fish-wife. But when an educated man and a gentleman stands up and states deliberately and in cold blood the very same things that the fish-wife uttered in her rage, the case assumes a different complexion. Similarly, there are newspapers that are responsible and newspapers that are irresponsible. When a scurrilous print in an obscure American town shrieks itself hoarse with pretended rage, or triumphantly proclaims that it has invented a bigger lie than any of its contemporaries—that is one thing. But the *Pall Mall Gazette* has a history; it has had a severe and honourable Morley as one of its editors, and an enthusiastic and justice-loving Stead as another. When a newspaper like this says that a whole class of public officials has shown "a blind and fatal obscurantism" in the conduct of their work, and that the phrase "More Hospital Scandals," has become a "familiar heading in the Press," we are brought to a standstill. This must be challenged. If the editor of the *Pall Mall* in his private capacity, were to make an accusation of so injurious a kind as this to the face of any particular hospital official, that official would be justified in taking him by the throat and in compelling him either to substantiate his words or to acknowledge that he had committed a serious offence, and one that he must, as a gentleman, undo, and make an ample apology for.

The Press of England has a character to maintain, exactly as every honourable gentleman in England has a character to maintain. No writer who has a due regard for his honour will say in his paper what he would not say in his private capacity. Is it not clear then that the hospital officials have a right to demand that the editor of the *Pall Mall Gazette*, as its responsible head, shall either prove the truth of his statements as publicly as he has made them; or that he shall withdraw them, also as publicly, and make a gentleman's apology?

In the meantime, those of our readers who desire to act fairly by the hospitals, will perhaps, consider what is now to be said! And, in the first place, we affirm with all sobriety that there is not, taking the London hospitals as a whole, one shred of justification for the charges which the *Pall Mall Gazette* has made. Two circumstances are to be borne in mind here; first, that the voluntary hospitals of London are supported by the free gifts of certain classes of the people: secondly, that those people who give the money are the very people who, by themselves, or by their elected representatives, manage the affairs of the hospitals. Is it conceivable that the people who give the money will allow it to be wasted, or to be put to any but the best possible use?

There are are ample reasons for believing that the writer of the article in the *Pall Mall Gazette* knows practically no more about the affairs of the management of the London voluntary hospitals than he knows about the affairs and the management of the Greenland whale fishery, or of the manufacture of American pills. To the initiated his words are of less significance than a puff of wind, if the wind happenes to be cold and frosty. There is reason to believe that not only has he not taken the trouble to acquire any real knowledge of hospital affairs, but that he has ostentatiously avoided making use of opportunities when they have been put in his way.

But this is the writer who at Chistmas time stands, so to speak, at the doors of all theLondon hospitals, and says, in the loudest tones that he can command, these are "scandalous" places: they are conducted on "principles of blind and fatal abscurantism." Bring no money here: the work inside is worthy only of obloquy and reprobation. It is of no concern to an irresponsible person of this kind that the sick workman has practically no refuge in dangerous illness except the hospital, or the dreaded workhouse: it is no concern of his that the hospitals place at the service of the poorest of London's inhabitants the best skill of a thousand of the most competent physicians and surgeons in the world without the cost of a single penny to London's workmen: it is of no moment that nursing as careful and as kind as the Queen herself can command is there available. The writer in the *Pall Mall* recks nothing of all these things. So far as he has said anything to the contrary, half the hospitals in London may shut up their doors forthwith.

This is responsible journalism: this is English fairplay: this is the *Pall Mall Gazette's* "peace and good will" of Christmas time! Who will envy an editor who pens such a paragraph about our noble English charities and sends it abroad to the world! We make no appeal for the hospitals at the end of an article like this. If the history of their past fifty years of work does not speak for them to the heart of every right-minded Englishmen, then our countrymen of the present generation are not what we believe them to be; they are not worthy of the name and fame and charitable deeds of the England of their fathers.

We publish below a letter which has been sent to the *Pall Mall.* The members of the Press generally are appealed to, in the interests of the most important of all our charities, to make themselves acquainted with the facts, and to speak with authority on a question so absolutely vital to the working classes of London and the country.

A Blind and Fatal Obscurantism.

*To the Editor of the "Pall Mall Gazette."*

